# Hemorrhagic Fever With Renal Syndrome in Vladivostok City, Russia

**DOI:** 10.3389/fpubh.2021.620279

**Published:** 2021-02-05

**Authors:** Liudmila N. Yashina, John Hay, Natalia A. Smetannikova, Tatiana V. Kushnareva, Olga V. Iunikhina, Galina G. Kompanets

**Affiliations:** ^1^Department of Genomic Research, State Research Center of Virology and Biotechnology “Vector”, Koltsovo, Russia; ^2^Department of Microbiology and Immunology, Jacobs School of Medicine and Biomedical Sciences, University at Buffalo, Buffalo, NY, United States; ^3^Department of Microbiology and Virology, Pacific State Medical University, Vladivostok, Russia; ^4^Laboratory of Experimental Virology, Somov Institute of Epidemiology and Microbiology, Vladivostok, Russia

**Keywords:** hantavirus, Seoul virus, *Rattus norvegicus*, hemorrhagic fever with renal syndrome, Russia, Vladivostok

## Abstract

Hemorrhagic fever with renal syndrome (HFRS) is a public health problem in Vladivostok city, Russia. From 1997 to 2019, a study of hantaviruses in Norway rats (*Rattus norvegicus*), a natural reservoir of Seoul virus (SEOV), and in HFRS patients was conducted. We demonstrated the presence of SEOV in the local population of Norway rats and detected SEOV in 10, Amur virus (AMRV) in 4 and Hantaan virus (HTNV) in 1 out of 15 HFRS patients. Genetic analysis based on partial S, M and L segment sequences revealed that the Russian SEOV strains were related most closely to strains from Cambodia and Vietnam. We postulate that the SEOV strains found in the port city of Vladivostok have been spread from South-East Asia as a result of distribution of rats during standard shipping trade activities. Moreover, we suggest that city residents may have acquired AMRV and HTNV infection during visits to rural areas.

## Introduction

Hemorrhagic fever with renal syndrome (HFRS) is endemic around the world. The disease is caused by viruses belonging to the genus *Orthohantaviruses*, that includes Hantaan virus (HTNV), Seoul virus (SEOV), Dobrava/Belgrade virus (DOBV), Puumala virus (PUUV) and Tula virus (TULV) (1, 2). Hantaviruses (family *Hantaviridae*) possess a negative-sense, single-stranded, tripartite RNA genome, comprising L, M, and S segments, which encode an RNA-dependent RNA polymerase (RdRp), envelope glycoproteins (Gn and Gc) and a nucleocapsid (N protein), respectively. HFRS-associated hantaviruses are transmitted by aerosolized excreta of their natural hosts, rodents belonging to the Muridae and Cricetidae families.

SEOV is the only hantavirus with a worldwide distribution: Asia (South Korea, Japan, China, Indonesia, Cambodia, Singapore, Vietnam), both Americas (USA, Brazil, Argentina), Africa (Egypt), Eurasia (Russia), and Europe (France, Belgium, United Kingdom, the Netherlands and Sweden) (3). SEOV is carried by wild Norway rats (*Rattus norvegicus*) and is classified as causing mild to moderate clinical forms of HFRS. SEOV has also been detected in other species of rats, e.g., *Rattus rattus* and *Rattus losea* (4). Phylogenetic analysis has identified at least seven distinct genetic lineages of SEOV (5). It has been suggested that, unlike other hantavirus species, which followed natural on-land migrations of their hosts, the main reservoir of SEOV, Norway rats, is omnipresent due to global trade and human migration (6, 7). For example, SEOV strains from France and Belgium do not show geographic clustering but are closely related to strains from Vietnam, Cambodia, Singapore, and Indonesia (5, 8, 9).

Russia included HFRS in the official reporting system of the Ministry of Public Health in 1978, and 131,590 cases of HFRS have been registered from 2000 to 2017 (10). In European Russia, reported cases are caused by PUUV and two types of DOBV, Kurkino virus and Sochi virus. HTNV, HTNV-related virus Amur (AMRV), and SEOV have been identified by us as causative agents of HFRS in Far Eastern Russia (11). SEOV has been detected in wild rats in the Asian part of Russia, in Primorsky, Amursk, and Sakhalin regions (12, 13). However, human cases of SEOV infection have only been seen in Vladivostok city (Primorsky region) since 1992 (11). The virus in Vladivostok was shown to cause generally mild to moderate HFRS, while 5.7% cases had severe clinical manifestations (12). Serologic differential diagnosis based on neutralization tests have demonstrated that the major cause of HFRS in Vladivostok city is SEOV (14). The genetic evidence for SEOV infection in both rodents and humans in Vladivostok city and the high percent of SEOV-associated HFRS cases (>60%) showed the importance of this hantavirus for public health in this part of Russia (15). Here, we investigate SEOV infection in wild rats and characterize etiologic agents of HFRS among the citizens of Vladivostok during a period of 23 years.

## Materials and Methods

Norway rats were trapped throughout Vladivostok city according to well-established protocols (16). Hantavirus infection in rats was analyzed by detection of virus-specific antibodies (Ab) or/and hantavirus antigen (Ag). Sera were screened for IgG and/or IgM anti-hantavirus Ab by an indirect immunofluorescence assay (IFA) using Vero E6 cells infected with PUUV, HTNV and SEOV as antigens (17). Lung tissues were analyzed for whole hantavirus Ag by ELISA using “HANTAGNOST” kit (Russia).

Blood samples of suspected HFRS patients from Vladivostok city were collected between 1997 and 2019. Clinical diagnosis of HFRS was laboratory-confirmed by the presence of IgG and/or IgM anti-hantavirus Ab, detected by IFA using “HFRS diagnostic kit” (Federal State Unitary Enterprise on Manufacture of Bacterial and Viral Preparations of Chumakov Institute of Poliomyelitis & Viral Encephalitis). For each HFRS patient a 4-fold increase in antibody level over a 2-week interval was registered. For genetic analysis, moderate and severe clinical HFRS cases were selected. The clinical disease severity of HFRS patients was subdivided into mild, moderate, or severe following the standard Russian criteria (length of febrile phase, minimal blood pressure in the hypotonic phase, extent of hemorrhagic symptoms, minimal urine production, serum creatinine level, and extent of proteinuria) (18). Signed informed consent was obtained from each patient (article 20, Federal Law “Protection of Health Right of Citizens of Russian Federation” N323-FZ, 11.21.2011).

RNA was extracted from rat lung tissues and human blood clots using the RNeasy Mini Kit (Qiagen), and cDNA was synthesized using Revert Aid premium reverse transcriptase (Thermo Scientific). Samples were screened for hantaviruses by nested RT-PCR, targeting the conserved regions of the viral genome. Three sets of nested primers to recover partial S (nt 610-936), M (nt 2737-2980) or L (nt 175-511) segment sequences were used (Supplementary Table 1).

The distance-based neighbor-joining and maximum likelihood methods, supported by MEGA 5 (19), were used to construct phylogenetic trees.

## Results

During the period 1997–2019, 750 human HFRS cases were laboratory-confirmed in Vladivostok city. 24.4% patients had a mild clinical course, 62.7% were moderate, and 12.9% had severe forms of disease. HFRS incident rate varied from 4.2 to 8.4 per 100,000 population (Table 1). At the same time, active circulation of hantavirus was detected in urban population of *R. norvegicus*, the primary reservoir for SEOV. A total of 1,903 Norway rats were captured in Vladivostok city in 1997 and between 2000 and 2014 (Table 1). Hantavirus-infected animals were found every year. The overall infection rate of *R. norvegicus* varied from 2.0% in 2011 to 31.4% in 2000. The highest incident rate in humans and a high infection rate among rats was registered during 1997–2007.

**Table 1:** Annual dynamic of *Rattus norvegicus* infection rate and HFRS morbidity in Vladivostok city, Russia (1997–2019).

**Year**	**Number hantavirus positive/tested rats**	Infection rate^*^ in rats (%)	95% CI^**^	Incidence per 100,000 human population^***^	**Number HFRS patients**
1997	14/71	19.7	10.5–28.9	5.9	37
1998	–	–	–	8.4	53
1999	–	–	–	7.6	48
2000	22/70	31.4	20.6–42.2	7.7	48
2001	14/63	22.2	12.0–32.4	8.1	48
2002	41/406	10.1	7.2–13.0	6.9	41
2003	7/35	20.0	6.7–33.3	7.7	49
2004	4/16	25.0	3.8-46.2	6.4	38
2005	4/26	15.4	1.5–29.3	6.3	43
2006	24/277	8.7	5.4–12.0	4.9	30
2007	48/166	28.9	22.0–35.8	5.4	33
2008	11/83	13.3	2.1–24.5	5.0	31
2009	18/95	18.9	11.1–26.7	5.0	31
2010	13/168	7.7	3.7–11.7	5.0	31
2011	2/98	2.0	0–4.8	5.0	31
2012	5/132	3.8	0.5–7.1	3.5	22
2013	16/147	10.9	5.9–15.9	4.2	26
2014	10/50	20.0	8.8–31.2	4.8	30
2015	–	–	–	2.7	17
2016	–	–	–	2.1	13
2017	–	–	–	1.4	7
2018	–	–	–	3.9	22
2019	–	–	–	3.4	21
Total	253/1,903	13.3	11.8–14.8	5.3	750

* *Infection rates are number hantavirus antibody and/or antigen positive rats/number tested*.

** *CI - confidence interval*,

*** *incidence was calculated for current number of city residents (mean number= 601,500); “–” not tested*.

For molecular typing of Russian SEOV strains, lung samples of Ag-positive rats, captured in 1997 and 2006, and human blood samples from Ab-positive HFRS patients, infected in 2000–2001, 2004, 2008, 2011, 2018–2019 were tested. Samples from patients with moderate and severe clinical forms of HFRS were selected for analysis. Hantaviral RNA (328, 244, and 337 base pairs for S, M, and L segments, respectively) was identified in 4/4 rat samples and in 15/28 human samples (Table 2). All recovered viral sequences were deposited in GenBank (accession numbers: MW073474-MW073498, MW088971-MW088982).

**Table 2:** Hantavirus strains identified in *Rattus norvegicus* and HFRS patients from Vladivostok city, Russia.

**Virus**	**Strain**	**Source**	Clinical case^*^	**Year**	**S**	**M**	**L**
SEOV	Vlad18992/Rn/1997	*R. norvegicus*		1997	MW073474	MW073489	MW088971
	Vlad18995/Rn/1997			1997	MW073475	MW073490	MW088972
	Vlad28935/Rn/2006			2006	MW073476	MW073491	–
	Vlad29910/Rn/2006			2006	–	MW073492	MW088973
	Vlad127/HU/2000	Human	Severe	2000	–	–	MW088974
	Vlad220/HU/2000		Severe	2000	–	–	MW088975
	Vlad725/HU/2001		Moderate	2001	MW073477	MW073493	MW088976
	Vlad2464/HU/2004		Severe	2004	MW449254	MW073494	MW088977
	Vlad4341/HU/2008		Moderate	2008	MW073478	MW073495	MW088978
	Vlad5163/HU/2011		Severe	2011	MW073479	MW073496	MW088979
	Vlad7139/HU/2018		Severe	2018	MW073480	–	MW088980
	Vlad7259/HU/2018		Severe	2018	MW073481	MW073497	MW088981
	Vlad7352/HU/2019		Severe	2019	MW073482	MW073498	–
	Vlad7364/HU/2019		Severe	2019	MW073483	–	–
HTNV	Vlad7308/HU/2018		Severe	2018	MW073484	–	–
AMRV	Vlad7194/HU/2018		Severe	2018	MW073485	–	MW088982
	Vlad7274/HU/2018		Severe	2018	MW073485	–	–
	Vlad7300/HU/2018		Severe	2018	MW073487	–	–
	Vlad7487/HU/2019		Severe	2019	MW073488	–	–

“*–” not tested*,

* *clinical case for human HFRS patients*.

**Figure 1:**
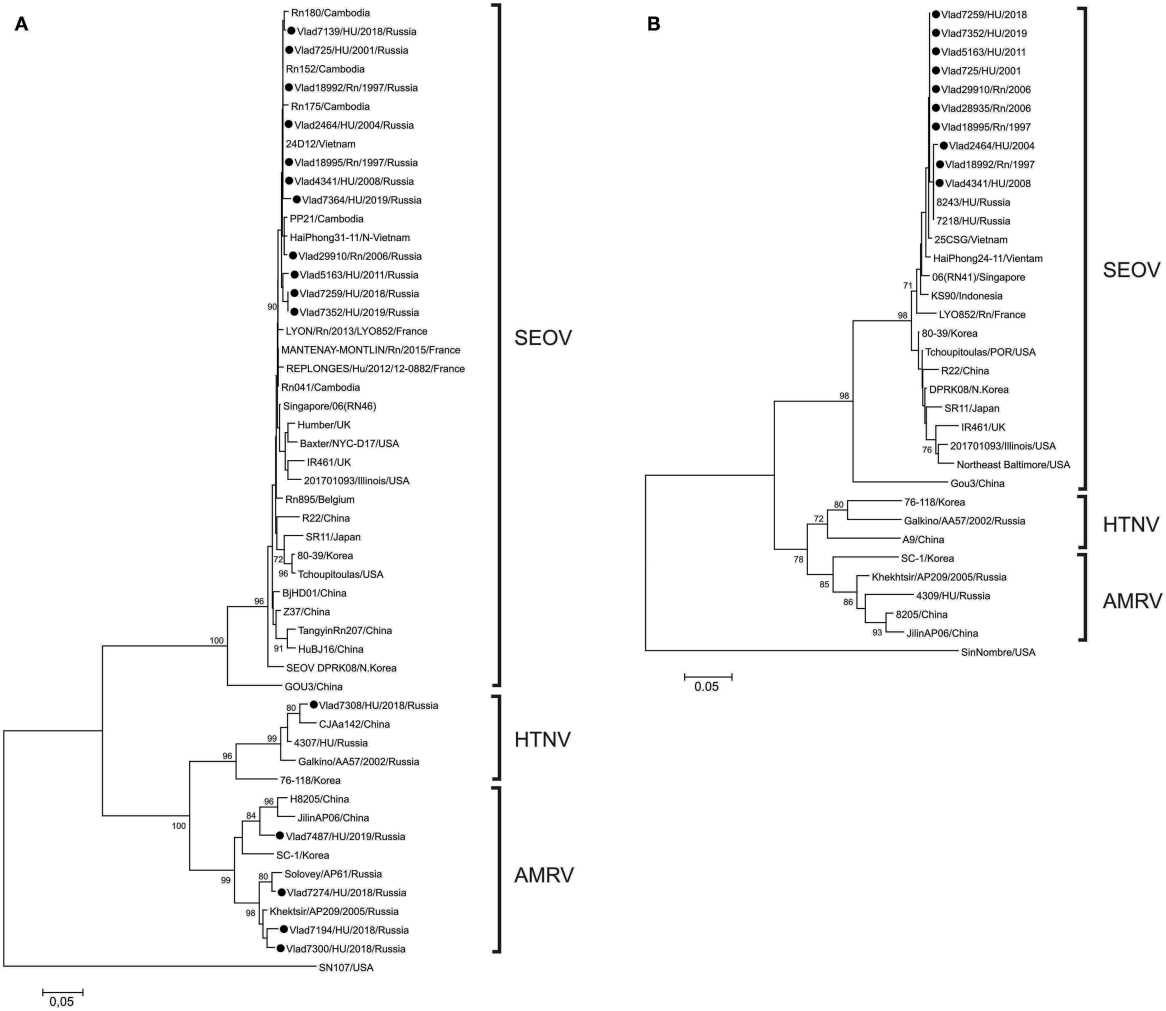
Phylogenetic trees of orthohantaviruses based on partial sequences of the **(A)** S (nt 610-936) and **(B)** M (nt 2737-2980) segment sequences. All analyses (neighbor-joining method) were performed using the MEGA software (19). Sin Nombre virus was used as the outgroup. Bootstrap values were calculated from 1,000 replicates; only values >70% are shown at the branch nodes. The trees constructed by using the maximum-likelihood method (not shown) had similar topology. Circles indicate sequences of strains detected in Norway rats and from the patients in this study.

Phylogenetic analysis of partial S and M segment sequences recovered from *R. norvegicus* showed similarity to that of the SEOV strains previously identified in HFRS patients and rats from Vladivostok city and also in rats from south-eastern Asia (Cambodia, Vietnam, Indonesia, Singapore) (Figure 1). Phylogenetic analysis of sequences recovered from HFRS patients identified three different hantaviruses: SEOV in 10/15, AMRV in 4/15, and HTNV in 1/15 samples. The nucleotide sequence divergence among the newly identified SEOV strains were 0–1.4%, 0–0.9%, and 0–1.9% for the S, M, and L segments, respectively, and deduced amino acid sequences were identical. Compared with previously published SEOV S and M nucleotide sequences from south-eastern Asia, divergence did not exceed 1.6%, while the differences with the SEOV strains from other regions (China, Korea, Japan, USA) reached 5.7%. Partial S segment sequences from two rats, captured in 1997, and three HFRS patients infected in 2001, 2004, and 2008 were identical to those of strains 24D12 from Vietnam and strain Rn152 from Cambodia (20, 21). The partial amino acid sequences of the RdRp, Gc, and N proteins of the SEOV strains from Vladivostok were identical with that of SEOV strains from south-eastern Asia and strains found in France, USA and Belgium.

Analysis of AMRV partial S segment sequences showed that patients from Vladivostok were infected by three genetic lineages of virus (Figure 1). Two of these lineages joined previously identified strains from far-eastern Russia and partial S segment divergence between sequences within each lineage did not exceed 5.2%. The third lineage was formed by a novel Russian strain and strains from the neighboring Jilin province of China.

As expected, the Vladivostok HTNV sequence was more closely related to HTNV strains from far-eastern Russia and the neighboring north-eastern area of China. The partial S sequence divergence between strain from Vladivostok and strains from north-eastern China (strains CJAa142, Fuyuan-Aa-26, Bao14, ShenyangAa13) was 2.7–4.3%. All AMRV- and HTNV-infected human patients had severe clinical manifestations of HFRS.

## Discussion

The results of genetic analysis of SEOV strains from rats and HFRS patients over two decades demonstrate the high stability of SEOV strains circulating in Vladivostok city. Phylogenetic analysis of the short sequences of SEOV that we obtained does not allow further differentiation of the lineages. However, the complete identity of the partial S segment sequences from Vladivostok with those from Cambodia and Vietnam for five strains isolated during 10 years leads us to define the origin of the virus and its phylogenetic placement into lineage 7, joining strains from Cambodia, Vietnam, Indonesia, Singapore and imported strains in France and Belgium (5, 8). In the Primorsky region, SEOV was detected in Norway rats and HFRS patients only in Vladivostok city, while circulation of a closely related genetic variant of SEOV among *R. norvegicus* was registered in large areas of South-East Asia, including the southern and central highland area of Vietnam, and the southern area of Cambodia. So, we suggest that SEOV in Vladivostok port city originated as a result of movement of its natural host from South-East Asia during intensive shipping trade activities between Vietnam, Cambodia and Russia.

Among serologically confirmed HFRS cases associated with SEOV, 31.5% patients had a mild clinical course and 62.8% were moderate, while 5.7% had severe forms of HFRS (12). Our direct genetic evidence shows that the SEOV strains from Vladivostok can cause severe clinical manifestations of HFRS. Also, some patients in Vladivostok were infected with AMRV and HTNV (4/15 and 1/15, respectively) and these finding are consistent with serologic data from a previous report (14). Phylogenetic analysis demonstrated that AMRV cases belong to three genetic lineages of AMRV. So, most probably, these HFRS patients were infected in different parts of the Primorsky region, likely located in the suburbs of Vladivostok city. City residents may acquire AMRV and HTNV infection from *Apodemus peninsulae* (a natural host of AMRV) and *A. agrarius* (a natural host of HTNV) during visits to their vegetable gardens in neighborhoods of Vladivostok or on trips to forests for nut harvesting and hunting.

In our study, a limited number of strains were genetically typed and information regarding the location of exposure for some patients from Vladivostok was not available. However, our data are in accordance with previously published results. In the Primorsky region, SEOV circulation was registered only in Vladivostok city but not in rural areas (12, 22). As the same genetically stable variant of SEOV, distinct from strains in neighboring China, was detected both in rats (4 strains) and human patients (10 strains) in Vladivostok, we inferred that patients who acquired this SEOV strain did so in the city. Identification of genetically divergent AMRV (4 strains) and HTNV (1 strain) from HFRS patients supports our hypothesis that some city residents likely acquired AMRV and HTNV infection from areas outside Vladivostok, where these strains circulate. Also, the proportion of severe HFRS cases (12.9%) among residents of Vladivostok that we detected, exceed those (5.7%) among serologically confirmed SEOV-associated patients, as published previously (12).

In summary, our study indicates that the main causative agent of HFRS in Vladivostok is SEOV, originating from infected Norway rats traveling by ship from south-east Asian countries. The other HFRS pathogens, AMRV and HTNV, showed clear spatial association with local geography.

## Data Availability Statement

The raw data supporting the conclusions of this article will be made available by the authors, without undue reservation.

## Ethics Statement

The studies involving human participants were reviewed and approved by Ethics committee of Somov Institute of Epidemiology and Microbiology, Vladivostok, Russia. The patients/participants provided their written informed consent to participate in this study. Ethical review and approval was not required for the animal study because Wild Norway rats were trapped according to well-established protocols (16).

## Author Contributions

All authors listed have made a substantial, direct and intellectual contribution to the work, and approved it for publication.

## Conflict of Interest

The authors declare that the research was conducted in the absence of any commercial or financial relationships that could be construed as a potential conflict of interest.
