# Supplementary material for: Hemorrhagic Fever With Renal Syndrome in Vladivostok City, Russia
Source: Front Public Health. 2021 Feb 5;9:620279. doi: 10.3389/fpubh.2021.620279 (PMC7892620; doi:10.3389/fpubh.2021.620279)
Supplement: Supplementary file 1 [file Table_1.DOCX]

Supplementary Table. Oligonucleotide primers for nested RT-PCR used in the study

| Genomic region | Primer | Position^a^, polarity, sequence (5`-3`) |
| --- | --- | --- |
| M segment, G2 | HS1  HS2 | 2636(+) AC(A/C)TGTCA(C/A)TTTGG(A/T)GACCC  3072(-) TCACA(G/A)GCCTTTATTGA(G/T)GT |
|  | HS3  HS4 | 2715(+) T(T/C)AGGAA(GA)AAATG(TC)AACTTTGC  3000(-) ACACC(A/T)GAACCCCAGGC(A/C)CC |
| S сегмент, N | SH1  SH2 | 364(+)GCGATGA(G/A)CC(G/A)ACAGGACA(G/A)AC(A/T)GC  972(-)TGGTGC(T/C)CCAGCAAA(C/G)ACCCA |
|  | SH3  SH4 | 578(+)TGCC(A/C)AATGCACA(G/A)TC(A/T)AG(C/T)ATGAA  956(-)ACCCA(T/G)ATTGA(G/T)GATGGTGA(C/T)TC(A/G)AT |
| L сегмент, RdRp | LH1  LH2 | 28(+)GGGGAAAA(G/A)(G/A)ATGGA(T/G)AAATATAGAGAAAT  576(-)ACACCATCATT(T/C)CT(T/C)(G/C)TACT(T/G)GGCCA |
|  | LH3  LH4 | 147(+)ACATTGT(A/T)GACCA(A/G)ATGAT(A/T)AA(G/A)CATGA  532(-)GCCATCTGT(G/C)CG(A/G)ACTGCAAC |

^a^The numbers correspond to genome positions of HTNV, strain 76-118
